# Sleep Parameters in Short Daily versus Conventional Dialysis: An Actigraphic Study

**DOI:** 10.1155/2017/2473217

**Published:** 2017-08-17

**Authors:** Ludimila D'Avila e Silva Allemand, Otávio Toledo Nóbrega, Juliane Pena Lauar, Joel Paulo Russomano Veiga, Einstein Francisco Camargos

**Affiliations:** ^1^Universidade de Brasília (UnB), Brasília, DF, Brazil; ^2^Centro Brasiliense de Nefrologia, Brasília, DF, Brazil

## Abstract

Previous studies have observed worse sleep quality in patients undergoing conventional dialysis as compared to daily dialysis. Our aim was to compare the sleep parameters of patients undergoing daily or conventional dialysis using an objective measure (actigraphy). This cross-sectional study was performed in three dialysis centers, including a convenience sample (nonprobability sampling) of 73 patients (36 patients on daily hemodialysis and 37 patients on conventional hemodialysis). The following parameters were evaluated: nocturnal total sleep time (NTST), expressed in minutes; wake time after sleep onset (WASO), expressed in minutes; number of nighttime awakenings; daytime total sleep time (DTST), expressed in minutes; number of daytime naps; and nighttime percentage of sleep (% sleep). The Mini-Mental State Examination and the Beck Depression Inventory were also administered. The mean age was 53.4  ±  17.0 years. After adjustment of confounding factors using multiple linear regression analysis, no difference in actigraphy parameters was detected between the groups: NTST (*p* = 0.468), WASO (*p* = 0.88), % sleep (*p* = 0.754), awakenings (*p* = 0.648), naps (*p* = 0.414), and DTST (*p* = 0.805). Different from previous studies employing qualitative analysis, the present assessment did not observe an influence of hemodialysis modality on objective sleep parameters in chronic renal patients.

## 1. Introduction

The literature provides evidence that chronic diseases may compromise the quality of sleep. One example is end-stage renal disease (ESRD), in which 80% of affected individuals experience insomnia and other sleep disturbances [1, 2]. Additionally, an independent association has been observed between ESRD and reduced total and REM sleep times, possibly as a result of uremia, fluid overload, or both [3].

The clinical management of ESRD relies on renal replacement therapy through dialysis. Conventional dialysis, the most traditional modality, involves thrice weekly sessions lasting up to 4 hours. Recent studies have shown significant improvement in survival with the use of high-flux membranes, ultrafiltration control, bicarbonate dialysate, and other more efficient water treatments [4]. These advancements have led to the introduction of daily dialysis that has some advantages over conventional dialytic therapy, including shorter daily treatment times. On the other hand, the study of Locatelli and coworkers showed that only a subset of patients with low serum albumin experience increased survival [5]. Daily hemodialysis is indeed associated with better control of blood pressure, ventricular hypertrophy, depression, cognitive function, and also survival. The mechanisms are probably related to a decreased ultrafiltration rate and better volume control and not the use of high-flux membranes. Daily dialysis is provided five to seven times per week, with sessions lasting 1.5 to 2.5 hours per day. Improvements in clinical parameters in daily dialysis have been cited, such as treatment tolerance, blood pressure control, nutritional status, and adverse events (anemia and hospital admissions, among others), with increase in survival and quality of life as well as patient well-being [6].

Enhancement in sleep quality, which is often poor in individuals on maintenance hemodialysis, has also been suggested in association with daily dialysis, although the findings are inconclusive. A study performed by Jaber et al. [7] with individuals undergoing short daily hemodialysis (6 times a week) has shown significant improvement in the prevalence and severity of restless legs symptoms after 12 months. Sleep disturbances, assessed with a self-administered survey, were also significantly improved. In addition, a recent study comparing polysomnographic parameters of 15 patients on daily hemodialysis versus 15 patients on conventional hemodialysis did not detect significant differences in the prevalence of obstructive sleep apnea between the groups (33.3% for daily dialysis versus 53.3% for conventional dialysis, *p* = 0.08) [8]. Similarly, a study using the Medical Outcomes Study Sleep Problems Index II instrument did not detect statistically significant differences between frequent versus conventional dialysis in sleep quality at 12 months [9].

Thus, the aim of the present study was to compare the sleep profile of patients undergoing daily or conventional dialysis using an objective measure (actigraphy). We hypothesized that sleep parameters, especially total nocturnal sleep time, would be more favorable with daily dialysis.

## 2. Methods

### 2.1. Setting

The present cross-sectional study included nonprobability sampling of ESRD patients undergoing daily or conventional dialysis in three facilities in Brasília, Brazil. The first facility (*Centro Brasiliense de Nefrologia* (CBN)) is a private clinic providing daily dialysis (1.5 to 2.5 h sessions) exclusively to private insurance patients. The second facility, located in a university hospital (*Centro de Diálise do Hospital Universitário de Brasília* (HUB/UnB)), provides conventional dialysis (3 to 4 h sessions) exclusively to patients from the public health care system (Unified Health System (SUS)). The third facility (*Nephron-Unidade de Diálise*) provides care to both private and public health care patients, with focus on conventional dialysis (sessions lasting 3 to 4 hours). Baseline data were collected from medical records. The study was approved by the Research Ethics Committee at the School of Medicine, Universidade de Brasília (protocol CAAE 31399514.9.0000.5558). All participants included in the final sample signed an informed consent form before the procedures. All procedures performed in studies involving human participants were in accordance with the ethical standards of the institutional and/or national research committee and with the 1964 Declaration of Helsinki and its later amendments or comparable ethical standards.

### 2.2. Participants

We included community-dwelling patients aged ≥18 years with a confirmed diagnosis of ESRD, receiving hemodialysis treatment for at least 3 months, and living in the same address for the study period. Patients who were not capable of understanding or answering questionnaires were not included, as well as those with movement disorders, upper limb paralysis that could compromise actigraphy, psychiatric disorders or disabling cognitive disorders, history of traumatic brain injury with residual neurological deficit, or any other detectable unstable medical condition that could prevent the patient from completing the research protocol.

All interviews were performed by the principal investigator (Ludimila D'Avila e Silva Allemand) on two different occasions during hemodialysis sessions. Data were collected between October 2014 and April 2016.

### 2.3. Measurements

A brief questionnaire was administered for collection of demographic clinical data: age (years), sex (m/f), body mass index (BMI; kg/m^2^), schooling (years), income, ESRD etiology, duration of dialysis (hemodialysis vintage, in months), and dialysis shift (morning/afternoon/night). The use of hypnotics, antidepressants, or other psychotropic medications was investigated (yes/no), as well as tobacco and alcohol consumption (yes/no). Medical records were reviewed to obtain the most recent data on hemoglobin (g/dL), iron (*μ*g/dL), ferritin (*μ*g/L), phosphorus (mg/dL), calcium (mg/dL), albumin (g/dL), potassium (mmol/L), and pre- and postdialysis urea (mg/dL) levels. The following tests were performed in all patients: the Mini-Mental State Examination (MMSE) (cognitive profile) [10] and the Beck Depression Inventory (BDI-II) [11].

Sleep parameters were assessed using an actigraph (Actiwatch, Respironics, Inc., Mini-Mitter, Bend, OR) and a data analysis software platform (Actiware, version 5.59.0015, 2010). The following parameters were used: (1) wake threshold selection = medium; (2) wake threshold value = 40; and (3) sleep interval detection algorithm = 10 immobile minutes for sleep onset and sleep end.

The actigraph was placed on the arm without arteriovenous fistula for continuous monitoring over nine days. To reduce the possibility of bias, the first and last days were disregarded, and data referring to 7 days and 7 nights were analyzed. Patients kept a sleep diary to document the time at which they went to bed and turned off the lights, as well as any occasions when they had to remove the device. The diary was used for adjustment of actigraphy data if necessary. Participants also used the “event” marker in the actigraph to record the time getting into bed and out of bed. Actigraphy data for each participant were extracted after 9 days. Data were analyzed in a blind fashion, with the evaluator blinded to the type of dialysis.

The following outcomes were evaluated: nocturnal total sleep time (NTST), expressed in minutes; wake time after sleep onset (WASO), that is, the sum of wake times from sleep onset to final awakening, expressed in minutes; number of nighttime awakenings from sleep onset to final awakening (awakenings); daytime total sleep time (DTST), expressed in minutes; number of daytime naps (naps), that is, daytime sleep periods lasting more than 10 minutes; and nighttime percentage of sleep (% sleep) from sleep onset to final awakening.

### 2.4. Statistical Analysis

Student's *t*-test was used to compare continuous variables with close-to-Gaussian distribution in both treatment groups. The Mann–Whitney test was used for variables with non-Gaussian distribution regardless of treatment modality. Pearson's chi-square test was used to compare proportions between treatments.

The influence of potential confounding factors was tested by multiple linear regression models having sleep parameters (NTST, WASO, % sleep, awakenings, naps, and DTST) as dependent variables. Potential confounders, sex, age, schooling, income, hemodialysis duration and shift, use of medications, tobacco, and alcohol, and MMSE, BDI-II, and BMI scores, were entered into the model in a stepwise manner. The contribution of each variable to the model was estimated and compared according to specified entry or removal criteria. Independent variables were kept in the model if *p* = 0.15. A *p* < 0.05 was established as limit for significance. Statistical analyses were carried out using SAS 9.4 software (SAS Institute, Cary, USA).

## 3. Results

Of 80 eligible patients, six refused to participate (4 patients on daily dialysis and 2 patients on conventional dialysis) and one patient died. Thus, the final sample included 73 patients (36 patients on daily hemodialysis and 37 patients on conventional hemodialysis).

The mean age of participants was 53.4 ± 17 years (59.3 years for daily hemodialysis and 47.5 years for conventional hemodialysis, *p* = 0.002); 40 patients were male and 33 were female.  Table 1 describes the study population. ESRD was caused by hypertensive nephropathy (*n* = 24, 32.8%), diabetic nephropathy (*n* = 13, 17.8%), glomerulonephritis (*n* = 8, 10.9%), polycystic kidney disease (*n* = 8, 10.9%), lupus nephritis (*n* = 3, 4.1%), and other causes (*n* = 15, 20.5%).

There was no statistical difference between the groups in actigraphy parameters. Mean NTST, WASO, % sleep, awakenings, naps, and DTST were similar in the daily dialysis and conventional dialysis groups even after adjustment of confounding factors using multiple regression analysis (Table 2). However, when considering the overall sample (*n* = 73), multiple linear regression analysis showed that dialysis shift (morning, afternoon, or night) significantly influenced specific sleep variables. Patients undergoing hemodialysis in the afternoon or night had an additional 40.3 minutes of NTST as compared to patients undergoing dialysis in the morning (95% CI: 3.56–77.09; *p* = 0.032); and patients undergoing dialysis in the morning had a mean of 69 additional minutes of DTST as compared to patients undergoing hemodialysis in the afternoon or at night (95% CI: 25.59–112.33; *p* = 0.002).

BDI-II scores were negatively associated with WASO. Patients with lower BDI-II scores (higher total scores indicate more severe depressive symptoms) had higher mean WASO (95% CI: −2.25–−0.11; *p* = 0.030). In addition, patients using antidepressants had an additional 34 minutes of WASO and 8 additional awakenings as compared to patients who were not using antidepressants (95% CI: 17.75–49.96; *p* < 0.0001).

## 4. Discussion

In this sample of 36 patients undergoing daily dialysis and 37 patients undergoing conventional dialysis, no differences were observed between the groups in terms of objective sleep parameters. To the best of our knowledge, there are no available comparative studies employing objective parameters to assess sleep in patients with this profile. To date, studies have employed qualitative, nonparametric measures or overnight polysomnography [8, 9, 12].

Previous studies have demonstrated that actigraphy is sensitive to detecting sleep patterns associated with specific sleep disorders as well as with other medical or neurobehavioral disorders [13]. A high correlation has also been reported between the gold standard polysomnography and actigraphic estimates of total sleep time and sleep efficiency, with 88% accuracy of actigraphy to distinguish sleep from wakefulness [14].

The present study corroborates frequent reports from the literature which describe poor sleep quality in hemodialysis patients, as reflected in our sample by an overall mean NTST of 5.8 hours. A systematic review of the literature has shown high prevalence of sleep disturbances in ESRD patients on conventional dialysis [15]. Bastos et al. have also reported poor sleep quality (Pittsburgh Sleep Quality Index ≥ 6) in 75% of conventional dialysis patients (*n* = 100) [16]. However, few studies have addressed the impact of daily hemodialysis on sleep parameters. Most studies have focused on conventional dialysis, peritoneal dialysis, or nocturnal hemodialysis.

Elias et al. [3] have compared polysomnographic parameters in 15 patients on daily dialysis versus 15 patients on conventional dialysis strictly regarding the presence of obstructive sleep apnea (OSA). The difference in OSA prevalence between the groups reached only borderline significance (33.3% for daily dialysis and 53.3% for conventional dialysis, *p* = 0.08). That study did not compare variables such as sleep efficiency, daytime sleepiness, naps, or total sleep time, providing only evidence of associations between OSA and both low dialysis dose and poor cardiovascular outcomes.

Another study has been recently performed to compare the effect of daily and nocturnal dialysis versus conventional dialysis on self-reported sleep quality [8]. A standardized questionnaire was used (Medical Outcomes Study Sleep Problems Index II) to evaluate sleep disturbances (scored from 0 to 100, with higher value indicating poorer quality of sleep). The authors did not find statistically significant differences between the two dialysis modalities in terms of sleep quality at 12 months.

Sabbatini et al. [17] investigated whether the technical and therapeutic advancements of hemodialysis had any impact on sleep disturbances in 694 patients. A (nonvalidated) questionnaire was used, in addition to clinical data obtained from the medical chart, dialysis data, and lifestyle information. The authors observed that 86% of patients presented some sleep disturbance (nocturnal awakening in 92%, difficulty falling asleep in 67%, and early waking in the morning in 62%); insomnia was detected in 45%. That study also associated the presence of insomnia with longer duration of dialysis (>12 months) in patients undergoing dialysis in the morning and in those with high levels of parathyroid hormone.

Similarly, studies have demonstrated excessive daytime sleepiness in chronic dialysis patients (about 30%) [18, 19]. In our sample, actigraphy also revealed increased daytime sleepiness. DTST was about 3 hours per day in both groups, which could explain why patients take naps during dialysis sessions. Nevertheless, this finding may be overestimated, since actigraphy records body movements and the arm is often at rest during dialysis sessions.

The findings of the present study regarding depressive symptoms deserve special attention. In our sample, patients with fewer depressive symptoms according to the BDI-II had worse nocturnal sleep quality, represented by higher WASO. This disagrees with the report by Trbojevic-Stankovic et al. [20] who observed that individuals with depression undergoing dialysis had significantly worse sleep quality than nondepressed individuals. However, that study analyzed sleep quality (by the Pittsburgh Sleep Quality Index) as a parametric variable, which is not methodologically adequate. The fewer depressive symptoms detected in our daily hemodialysis group [versus conventional hemodialysis group] might be explained by the significantly higher use of antidepressants in this group.

It is possible that sleep disturbances may be related to other aspects beyond the renal/dialytic component. It is likely that the etiology of sleep disorders in these patients has a multifactorial nature [21]. Aspects linked to social and work activities (which are restricted in individuals undergoing dialysis), genetic and psychological factors, and lifestyle habits may interfere with sleep [22, 23]. However, other factors, which have not often been evaluated, may also be related to sleep disorders in these patients, such as anemia, levels of urea and uremic toxins in blood, cardiovascular diseases, arterial hypertension, diabetes, advanced age, time since the onset of dialysis, alcohol and tobacco abuse, and depression. As previously mentioned in the literature, a plausible cause might be the influence of biochemical parameters, such as hemoglobin, urea, and phosphorus levels [23]. In clinical practice, increased levels of phosphorus may be associated with bone pain, pruritus, and, consequently, sleep fragmentation.

Variations in urinary volume during sleep, associated with reduced sodium, calcium, and potassium excretion, may also be associated with sleep disorders, which are frequent in ESRD [24]. In addition, it is known that ESRD is associated with increased inflammation, which, according to some authors, could also influence sleep quality in these individuals [25].

Another important aspect in the evaluation of sleep in chronic renal patients is related to the shift during which the treatment is usually performed. Some studies have demonstrated that morning dialysis is significantly associated with worse sleep quality; it is possible that, beyond behavioral factors that may disturb sleep, such as early waking in the morning, metabolic factors may also affect the circadian rhythm [17, 21, 25]. A better quality of sleep has been observed after nocturnal dialysis [26]. Conversely, the patients in the present study who underwent morning dialysis had less nocturnal sleep and more daytime sleep as compared to those undergoing dialysis in the afternoon or at night. One hypothesis that could explain this finding is that the need to wake up very early for treatment sessions affects total sleep time, with an increase in the number of naps during the day.

Some methodological limitations of the present study must be addressed. First, the older age of patients on daily hemodialysis (59.3 years versus 47.5 years for participants on conventional dialysis) may have counterbalanced the worse biochemical parameters of patients on conventional dialysis, despite the statistical adjustment. Nevertheless, Sabry et al. [27] have identified high prevalence of sleep disorders in a sample of relatively young patients. Also, the cross-sectional design has limitations in and of itself; an observational study comparing sleep parameters before and after the onset of dialysis (daily and conventional) would allow assessment of other variables along the study period and enable the establishment of individual baseline parameters. Finally, pain, an important variable to be considered for the evaluation of sleep quality, was not addressed in this study. Davison and Jhangri have pointed out that, regardless of treatment modality, about 50% of dialysis patients experience pain and that those with moderate or severe pain experience insomnia more than those reporting mild or no pain do [28].

In summary, contrary to previous studies employing qualitative methods, the present study using actigraphy did not observe differences in sleep parameters between patients undergoing daily or conventional dialysis. Additional research is necessary to elucidate factors that may impair sleep in this population and promote better sleep quality for patients undergoing any of the currently available renal replacement modalities.

## Figures and Tables

**Table 1:** Demographic and clinical variables of patients undergoing dialysis  (*n* = 73).

Variable^*∗*^	Daily hemodialysis	Conventional hemodialysis	*p* ^#^
	*n* = 36	*n* = 37	
Age (years)	59.3 ± 18.6	47.6 ± 13.3	0.002
Sex			0.044
Male	24 (66.7)	16 (43.2)	
Female	12 (33.3)	21 (56.8)	
Schooling (years)	2.7 ± 0.5	1.6 ± 0.6	<0.0001
Hemodialysis vintage (months)	50.9 ± 38.5	59.2 ± 50.3	0.650
Treatment shift			0.074
Morning	22 (61.1)	27 (73.0)	
Afternoon	9 (25.0)	10 (27.0)	
Night	5 (13.9)	0 (0.0)	
Use of hypnotics			0.091
Yes	8 (22.2)	3 (8.1)	
No	28 (77.8)	34 (91.9)	
Use of antidepressants			0.0004
Yes	16 (44.4)	3 (8.1)	
No	20 (55.6)	34 (91.9)	
Other psychotropic medications			0.590
Yes	6 (16.7)	8 (21.6)	
No	30 (83.3)	29 (78.4)	
Use of tobacco			0.674
Yes	3 (8.3)	2 (5.4)	
No	33 (91.7)	35 (94.6)	
Use of alcohol			0.188
Yes	8 (22.2)	4 (10.8)	
No	28 (77.8)	33 (89.2)	
Scales			
MMSE	27.9 ± 1.9	27.6 ± 2.2	0.378
BDI-II	10.6 ± 7.0	14.6 ± 6.9	0.013
Body mass index (kg·m^2^)	25.1 ± 5.0	25.7 ± 3.8	0.577
Hemoglobin (g/dL)	11.7 ± 1.3	10.9 ± 2.0	0.071
Iron (*µ*g/dL)	62.9 ± 28.7	64.4 ± 26.1	0.742
Ferritin (ng/mL)	331.2 ± 326	379.2 ± 275	0.499
Albumin (g/dL)	4.0 ± 0.6	4.0 ± 0.3	0.563
Predialysis urea level (mg/dL)	107.8 ± 34.9	132.6 ± 34.5	0.003
Postdialysis urea level (mg/dL)	51.1 ± 19.0	35.4 ± 17.7	0.0005
Potassium (mEq/L)	5.0 ± 0.7	5.4 ± 0.9	0.031
Phosphorus (mg/dL)	5.4 ± 1.5	5.5 ± 1.5	0.725
Calcium (mg/dL)	9.1 ± 0.8	9.0 ± 0.7	0.393

^*∗*^Mean  ± standard deviation or frequency (%). ^#^Student's *t*-test, Mann–Whitney test, or Pearson's chi-square.

**Table 2:** Actigraphic variables, sleep quality, and daytime sleepiness in patients undergoing two modalities or hemodialysis (*n* = 73).

Variable	Hemodialysis^*∗*^		*p* value^#^
	Daily (*n* = 36)	Conventional (*n* = 37)	
NTST (min)	348.0 ± 95.2	349.9 ± 63.9	0.468
WASO	76.0 ± 41.1	66.4 ± 33.4	0.188
% sleep	82.5 ± 8.4	84.8 ± 7.1	0.754
Awakenings (number)	26.8 ± 10.2	23.6 ± 8.1	0.648
Naps	43.6 ± 18.1	38.1 ± 15.7	0.414
DTST	188.9 ± 99.7	175.2 ± 93.7	0.805

^*∗*^Mean  ± standard deviation or frequency (%). ^#^Linear or multiple regression models adjusted for age, dialysis shift, use of hypnotics, use of antidepressants, and BDI-II score. NTST: nocturnal total sleep time; WASO: wake time after sleep onset; DTST: daytime total sleep time.
